# Vitamin D, Gut Microbiota, and Cardiometabolic Diseases—A Possible Three-Way Axis

**DOI:** 10.3390/ijms24020940

**Published:** 2023-01-04

**Authors:** Ayah Sukik, Joud Alalwani, Vijay Ganji

**Affiliations:** Department of Human Nutrition, College of Health Sciences, QU Health, Qatar University, Doha P.O. Box 2713, Qatar

**Keywords:** 25-hydroxyvitamin D, diabetes, gut microbiome, metabolic syndrome, microbiota, obesity, type-2 diabetes, vitamin D

## Abstract

Metabolic syndrome (MetSyn) is a precursor for several cardiometabolic diseases such as obesity, type-2 diabetes mellitus (T2DM), and cardiovascular diseases. Emerging evidence suggests that vitamin D deficiency links to cardiometabolic diseases through microbiota. A combination of poor vitamin D status and dysbiosis may contribute to the progression of cardiometabolic diseases. Therefore, in this review, we present the relationship among vitamin D, microbiota, and cardiometabolic diseases with a focus on MetSyn. We searched major databases for reports on vitamin D, microbiota, and MetSyn until June 2022. We reviewed 13 reports on the relation between vitamin D and MetSyn (6 randomized controlled and 7 cross-sectional studies) and 6 reports on the effect of vitamin D on the gut microbiome. Adequate vitamin D status has a beneficial effect on gut microbiota, therefore preventing the progression of MetSyn. Further, well-controlled studies are needed for a better understanding of the mechanisms of action involving vitamin D and microbiota in the pathogenesis of cardiometabolic diseases.

## 1. Introduction

Vitamin D, a lipophilic vitamer, is synthesized in the skin upon exposure to the sun’s UV light in addition to obtaining it through limited dietary sources [1]. The dietary and the endogenously synthesized vitamin D are inactive until they undergo two hydroxylation reactions, one in the liver [vitamin D to 25-hydroxyvitamin D (25(OH)D)] and the second one in the kidneys [25(OH)D to 1,25-dihydroxyvitamin D (1,25(OH)_2_D)]. The active, hormone form is 1,25 (OH)_2_D. The major circulatory form and the widely used marker of vitamin D status is 25(OH)D. It circulates by binding to vitamin D binding protein (VDB) [2]. Further, 1,25(OH)_2_D binds to vitamin D receptors (VDR) which induce the gene expression of various proteins which in turn these proteins maintain calcium and phosphorus homeostasis in concert with the parathyroid hormone [3]. This is considered a classical function of vitamin D. Other non-bone functions of vitamin D are the regulation of cell proliferation, cell differentiation, apoptosis, and immunomodulation [3].

Metabolic syndrome (MetSyn), one of the cardiometabolic ailaments, is a group of derangements that include increased central adiposity, elevated glucose and blood pressure, and dyslipidemia (decreased HDL cholesterol and increased triglycerides) [4,5]. Globally, the prevalence of MetSyn ranges from <10% to 84%, depending on the geographical location, the composition of the studied population, and the criteria used in defining MetSyn [5]. There are a few differences in the criteria of MetSyn. For instance, central adiposity in men according to the World Health Organization definition requires BMI > 30 kg/m^2^ or a waist-to-hip ratio >9, whereas waist circumference is required by the National Cholesterol Education Program and International Diabetes Federation.

Recent evidence links vitamin D with several non-bone ailments such as MetSyn, obesity, type-2 diabetes mellitus (T2DM), and cardiovascular diseases (CVD) [6,7,8,9]. The exact mechanism through which vitamin D ameliorates pathologies associated with these diseases is not clearly understood. However, it has been proposed that vitamin D functions in upregulating anti-inflammatory responses, stimulating insulin production, and improving insulin sensitivity [10,11]. Evidence supports that the gut microbiome has been also associated with inflammation of diabetes and CVD [12,13]. Therefore, it is reasonable to hypothesize that the gut microbiome acts as a central mechanism, explaining the associations between vitamin D and cardiometabolic diseases. To our knowledge, this is the first review to evaluate the relationship between vitamin D and microbiota status, and their associations with MetSyn. In this review, we offer insights into the association and pathophysiological mechanisms linking vitamin D and microbiota with the risk of developing MetSyn and other cardiometabolic diseases.

## 2. Literature Search Strategy

PubMed, SCOPUS, and Google Scholar databases were searched for reports until June 2022 using the following terms: “vitamin D” or “25-hydroxyvitamin D” AND “metabolic syndrome” OR “cardiometabolic*”, and “microbiota*” AND “metabolic syndrome” OR “cardiometabolic*”. We have explored our proposed association by looking at diverse levels of evidence as discussed successively.

## 3. Vitamin D and Cardiometabolic Diseases: Proposed Mechanism of Action

Although a few mechanisms have been proposed for the role of vitamin D in MetSyn based on various levels of evidence, however, a clear picture is yet to emerge. Generally, persons with obesity have often reduced circulating vitamin D because it gets sequestered in adipose tissue. Therefore, obesity is an important determinant of low serum vitamin D [14]. The main contributor to pathologies of MetSyn is abdominal obesity [15,16]. Abdominal obesity is a risk factor for insulin resistance, which in turn negatively affects glucose homeostasis. This deranged glucose metabolism is a hallmark of MetSyn [17]. The molecular mechanism of vitamin D in glucose homeostasis appears to be multi-pronged. A function of vitamin D in glucose regulation has been realized after the VDRs were mapped in pancreatic β-cells [18]. Zeitz et al. found that VDR knock-out mice exhibited dysregulation of glucose and suppressed secretion of insulin [19]. Additionally, in adipocytes, calbindin is expressed in response to 1,25(OH)_2_D, a hormone form that enhances insulin action and promotes insulin section from β-cells of the pancreas [20,21]. Further, it was reported in obese mice that 1,25(OH)_2_D administration into the brain improved insulin sensitivity, improved blood glucose regulation, and reduced body weight and food intake [22]. Vitamin D decreases insulin resistance by the expression of insulin receptors and PPAR-γ, a nuclear receptor [23]. In vivo studies have shown that PPAR- γ regulates lipid metabolism and lowers MetSyn-associated dyslipidemia [24]. In addition, it has been proposed that hypovitaminosis D is associated with hyperlipidemia by reducing Sirtuin, a signaling protein involved in metabolic regulation. Sirtuin stimulates lipolysis and inhibits adipogenesis [25]. Another mechanism that relates hypovitaminosis D with MetSyn is vitamin D’s immunomodulatory and anti-inflammatory functions. Vitamin D mediates the stimulation of insulin production, improvements in insulin sensitivity, and regulation of the adaptive immune system through the inhibition of inflammatory cytokines production in T-cells, including down-regulation of pro-inflammatory cytokines such as TNF-α, IL1, IL6, and IL8 [8,11]. Despite strong evidence for cause and effect between vitamin D and insulin secretion, improved glucose regulation, lowered inflammation, and decreased insulin resistance, human vitamin D supplementation studies on CVD risk have resulted in mixed findings [26,27,28,29,30,31,32]

A blood pressure-lowering function has been attributed to vitamin D since VDRs, 1-α hydroxylase, and 1,25(OH)_2_D are mapped in endothelial cells [33]. Several mechanisms have been proposed to relate vitamin D deficiency and blood pressure. These include disruption in renin gene expression, and altered vascular tone through direct or indirect dysfunction of the endothelial and vascular smooth muscle cells [9,11]. VDR knockout mice exhibited decreased relaxation of blood vessels [34]. Further, vitamin D functions in blood pressure regulation via the action of the renin-angiotensin-aldosterone system. VDR knock-out mice had increased blood pressure owing to the elevated renin and angiotensin II concentrations [35,36]. Despite these findings related to vitamin D and MetSyn components, mixed results were reported in the literature [15,37]. Much confounding could be the reason for these disparate results.

### 3.1. Vitamin D and Cardiometabolic Diseases: Evidence from Interventional Studies

Interventional trials on the effect of vitamin D supplementation on cardiometabolic biomarkers have not yielded positive findings (Table 1). In a population with polycystic ovarian syndrome and vitamin D deficiency, a double-blind, randomized clinical trial (RCT) has shown that vitamin D supplementation (3200 IU/day) for 3 months did not influence cardiometabolic markers such as adiposity, glucose, blood pressure, insulin, and lipid profile (triglycerides and HDL-cholesterol) as compared to placebo [38]. In another 8-week clinical trial on clozapine-treated patients with chronic schizophrenia, no significant difference in metabolic parameters was observed between the vitamin D intervention group and placebo [39]. In Chinese participants with hypovitaminosis D and MetSyn, no difference was observed as compared to placebo in terms of their metabolic parameters post 12 months of vitamin D supplementation (700 IU/day). In this study, subjects had normal serum 25(H)D concentrations [40]. The lack of an association between vitamin D supplementation and metabolic biomarkers in these studies has been attributed to seasonal changes in long experiments resulting in daily and dietary habit variability contributing to differences in metabolic control, and seasonal changes resulting in reduced UV-light exposure in some seasons. Thus, influencing the endogenous production of vitamin D and the patient’s motivation for treatment may also confounded the results [40].

A few controlled studies found a beneficial effect of vitamin D supplementation on the markers of MetSyn. Cojic et al. in an RCT on the T2DM population treated with Metformin has shown that vitamin D supplementation resulted in a significant HbA1c reduction compared to placebo following a 3-month supplementation [8]. Although authors reported that vitamin D can stimulate insulin secretion through a calcium flux-dependent mechanism, however, results may have been confounded by the fact that those who received metformin were given lifestyle advice. Talaei et al. also observed that vitamin D supplementation (50,000 IU/week) for 8 weeks in subjects with T2DM decreased fasting blood glucose, insulin, and HOMA-IR significantly but did not affect lipid profile [41]. In another double-blind RCT, subjects who were on vitamin D supplementation (50,000 IU/week) for 4 months had significantly decreased triglycerides but had no effect on other cardiometabolic biomarkers [42]. The differences in results may be due to individual differences in VDR polymorphisms which may influence individuals’ responses to treatment [42]. Variability in vitamin D supplementation doses, variability in the length of studies, and differences in study populations are more likely reasons for the lack of a direct cause-effect relationship between vitamin D and MetSyn components in clinical trials.

### 3.2. Vitamin D and Cardiometabolic Diseases: Evidence from Epidemiological Studies, Systematic Reviews, and Meta-Analyses

Evidence from cross-sectional studies on the association between vitamin D and MetSyn is strong (Table 2). A cross-sectional study on the American population using the data from the National Health and Nutrition Examination Survey showed that the odds of having MetSyn were significantly (3 times) higher in the lowest serum 25(OH)D quartile compared to the highest quartile, emphasizing an inverse dose-response relationship between serum 25(OH)D and MetSyn [9]. In the PORMETSYN cross-sectional study, it was observed that increased serum 25(OH)D was associated with lower odds of elevated blood pressure and triglycerides [11]. Two cross-sectional studies conducted on the elderly reported that subjects who were hypovitaminosis D had a higher prevalence of MetSyn as compared to those who were vitamin D sufficient [10,15]. In disease-specific populations such as persons with non-alcoholic fatty liver disease, psychotic disorders, and systemic lupus erythematosus, low serum vitamin D was associated with MetSyn [43,44,45].

A significant association was observed in a dose-response fashion in a meta-analysis study based on cross-sectional studies. It was reported that an increment of 25 nmol/L in serum vitamin D was associated with a 19% lower risk of MetSyn in a set of cross-sectional and cohort studies, suggesting an inverse relationship between serum 25(OH)D and MetSyn [25]. A meta-analysis of 81 studies found vitamin D reduced blood pressure and blood lipids [46]. In contrast, Al Anouti et al. reported in their systematic review and meta-analysis of RCTs that correcting hypovitaminosis D through supplementation was not an effective intervention in improving dyslipidemia in individuals with MetSyn [16]. Although heterogeneity was high in these meta-analyses due to differences in study designs, subjects’ characteristics, and statistical analysis, the bias was low.

## 4. Microbiota and Cardiometabolic Diseases

The microbiota plays a role in a variety of functions impacting the physiology and pathology of the gut [47]. These functions include modulation of the host’s nutritional status, energy harvest, production of some vitamins (vitamin K, biotin, vitamin B-12, etc.) and fermentation of dietary fibers, intestinal epithelial homeostasis, the development of the host immune system, protection against pathogens, and drug metabolism [48]. Studies have shown that a persistent imbalance of the gut microbiota is related to diabetes, obesity, and CVD [49]. Among several factors, diet and dietary constituents play a vital role in maintaining and improving the beneficial microbiota [50,51].

The role of microbiota in MetSyn has been studied in animal models and humans [52,53,54,55,56,57,58,59,60,61,62,63] (Table 3). In basal conditions, the conventionally raised mice had a 40% higher body fat compared to germ-free mice and this phenomenon was independent of the food intake. After the transplantation of germ-free mice with microbiota from conventionally raised mice, a significant increase in body fat (60% increase) was observed in the germ-free mice [52]. Additionally, a significant increase in the synthesis of hepatic triglycerides and the development of insulin resistance were observed in the germ-free mice that received microbiota from conventionally raised mice. In another study, isolated Enterobacter cloacae B29 from the stool of obese subjects were transplanted into the germ-free mice [53]. As a result of this transplantation, the germ-free mice developed obesity and insulin resistance on a high-fat diet, but not on a normal diet. Germ-free control mice did not show the same metabolic effect on a high-fat diet after transplantation of Enterobacter from obese subjects. Enterobacter transplanted mice had high circulating endotoxin and inflammation. Based on these findings, it has been postulated that endotoxin derived from the bacteria in the gut is causatively related to insulin resistance and adiposity in humans.

In a human-animal study, fecal microbiota was transplanted from an adult female twin pair (one was obese and another was lean) into germ-free mice that were fed a low-fat chow, and other diets that contained diverse amounts of saturated fat, fruits, and vegetables. Mice that received a transplant from the obese twin donor had an increased total body weight, fat mass, and other metabolic features of obesity. Mice that received the microbiota from the lean twin did not show increased body mass or any obesity-associated metabolic features [54]. Further, Vrieze et al. performed microbiota transplantation from lean men to men with MetSyn [55]. The men with MetSyn experienced improved insulin sensitivity after 6 weeks of microbiota infusion. Based on these microbiota transplantation studies, it has been proposed that gut microbiota is involved in the regulation of human adiposity.

Studies relating the type of bacteria with adiposity have yielded equivocal findings. Ley et al. showed that the proportion of *Bacteroidetes* decreased in obese people in comparison to lean people [56]. This proportion increases with weight loss. In contrast to these findings, Duncan et al. reported that there were no significant differences in the B/F ratio between obese and lean subjects. Additionally, they observed, no significant changes in fecal *Bacteroidetes* count during the diet-induced weight loss [57]. In an animal study, mice that were fed a high-fat diet for 30 days had significantly increased colonies of *Firmicutes* and reduced colonies of *Bacteroidetes* [58]. Decreased ratio of B/F is related to increased energy harvest from food, increased adiposity, elevated inflammation, and increased insulin resistance, which are typical features of MetSyn.

The gut microbiome has also been found to be associated with diabetes in humans. A high-fat Western-style diet fed to individuals over one month induced a 71% increase in plasma concentrations of endotoxins (such as LPS), suggesting that endotoxemia may be a result of gastrointestinal barrier dysfunction associated with dysbiosis [59]. In contrast, a prudent diet reduced endotoxemia by 31%. In addition, similar findings were reported in animal studies. In a study, Cani et al. showed that mice that were fed a high-fat diet had an increased LPS in the gut and circulation [60]. Further, patients with T2DM have increased plasma LPS [61]. In a recent study, gut dysbiosis has been also associated with the risk of developing type I diabetes mellitus in children [62]. It is possible that diets high in fat cause dysbiosis, which leads to altered gut function leading to elevated LPS in circulation. These elevated bacterial products may influence the development of diabetes [63].

## 5. Proposed Mechanism of Action for Microbiota and Cardiometabolic Diseases

In humans, a primary effect of dysbiosis is the translocation of bacterial metabolites such as LPS, which induces chronic low-grade inflammation through the production of pro-inflammatory cytokines such as IL-1β [65,66]. As noted before, diet-induced dysbiosis has been observed in animal studies. Cani et al. [60] found that mice fed a high-fat diet had increased LPS-producing bacteria in the gut and 2 to 3 times higher LPS concentration in the circulation. Further, LPS subcutaneous infusion caused elevated fasted hyperglycemia, hyperinsulinemia, macrophage infiltration, weight gain, and insulin resistance [67]. These studies demonstrated the role of endotoxemia in the development of adiposity and diabetes.

A bacterial product known as 16S rDNA has been identified as a broad biomarker for diabetes and obesity. In a 9-year follow-up longitudinal, nested case-control study, Amar et al. measured 16S rDNA content at baseline and after a 9-year follow-up [64]. This study included subjects with diabetes and without both diabetes and obesity. They found that 16S rDNA concentration was higher in those who developed diabetes after the follow-up. The 16S rDNA content predicted diabetes in the multivariate-adjusted model (standardized OR 1.29; 95%CI, 1.08, 1.55).

## 6. Vitamin D and Microbiota in Cardiometabolic Diseases

**Figure 1:**
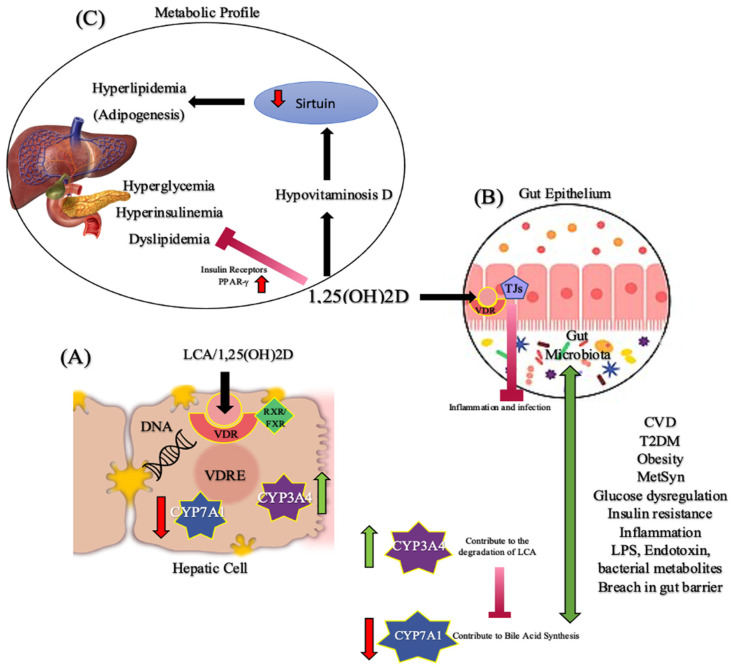
Vitamin D, gut microbiota, and cardiometabolic diseases–a possible three-way axis. (**A**) In the liver, LCA or 1,25(OH)_2_D activates VDR which in turn binds to the VDRE region of the DNA after heterodimerization with FXR/RXR. This activation results in the upregulating of CYP3A4 transcription which contributes to LCA degradation and the down-regulating of CYP7A1 transcription which contributes to bile acid synthesis/toxicity. (**B**) In addition, VDR is also activated in the gut epithelium. This activation results in the regulation of epithelial tight junctions that prevents any breach in the gut microbiota, thus preventing inflammation and pathogen infections while regulating secondary bile acids transformation by gut microbial metabolism. VDR plays a role in regulating this gut-liver axis. Any disruption in this axis will result in gut permeability, inflammation, MetSyn, and other cardiometabolic diseases. (**C**) 1,25(OH)_2_D has a multi-pronged independent action (that does not involve gut microbiota) that improves metabolic profile. Hypovitaminosis D is associated with hyperlipidemia by reducing Sirtuin, a signaling protein that is involved in metabolic regulation. Sirtuin stimulates lipolysis and inhibits adipogenesis. 1,25(OH)2D decreases insulin resistance by the expression of insulin receptors and PPAR-γ, a nuclear receptor. Abbreviations: 1,25(OH)_2_D, 1,25-dihydroxyvitamin D; CVD, Cardiovascular disease; CYP3A4, Cytochrome P450; CYP7A1, Cholesterol 7-α-hydroxylase; FXR, Farnesoid X receptor; LCA, Lithocholic acid; LPS, Lipopolysaccharide; MetSyn, Metabolic syndrome; RXR, Retinoid X receptor; T2DM, Type-2 diabetes mellitus; TJ, Tight junctions; VDR, Vitamin D receptor; VDRE, Vitamin D response element; PPAR-γ, Peroxisome proliferator-activated receptor gamma.

There is mounting evidence supporting the 3-way connection between vitamin D, microbiota, and cardiometabolic diseases. It is well-documented that gut microbiota plays an important role in the pathology of many chronic inflammatory diseases, such as obesity, diabetes, CVD, cancer, respiratory diseases, gastrointestinal diseases, psychological diseases, and a possible role in MetSyn [12,13,68,69,70]. Vitamin D activates genomic actions through VDRs. VDRs are activated in the liver by either 1,25(OH)_2_D or a secondary bile acid called lithocholic acid (LCA), which is produced by intestinal bacteria and acts as an additional physiological ligand for VDR. After activation, VDR heterodimerizes with FXR/RXR and then binds to the vitamin D-responsive element of DNA. Thus, VDR contributes to the degradation of LCA [71]. VDR activation also modulates the CYP7A1 gene which regulates the cholesterol conversion into bile acids [71]. Therefore, this gene decreases bile acid synthesis suggesting that VDR may act as a bile acid sensor. In the intestine, VDR has been found to maintain the intestinal barrier function by regulating epithelial tight junction proteins. This leads to the blocking of the translocation of bacteria or bacterial byproducts into the host’s circulation and reduces inflammation [71,72]. Thus, vitamin D plays a key role in linking VDRs with signaling pathways that moderate the gut-liver axis (Figure 1).

Further, VDRs have been shown to influence gut microbiota composition [73]. Studies have suggested that the lack of the VDR gene has a detrimental effect on friendly bacteria, such as Lactobacillus, which are known to have an anti-inflammatory effect. On the other hand, VDR knock-out mice had a higher risk for infections and inflammation compared to wild-type mice [73]. This highlights the restoring of VDR function to prevent exacerbated inflammatory status that may be involved in a higher risk of MetSyn through its interaction with gut microbiota [74].

Additionally, it has been shown that the low serum 25(OH)D is associated with increased LPS, which is involved in elevated inflammation and insulin resistance. This emphasizes the role of vitamin D in preserving immune homeostasis as a part of the integral relationship between gut microbiota and vitamin D [75]. A recent study on obese rats discussed the effect of vitamin D supplementation on gut microbiota. Rats received a high-fat, high-sucrose diet and either vitamin D (500 IU/kg/day) or metformin (200 mg/kg/day) for 8 weeks (about 2 months). Fecal, blood, and tissue samples were collected to examine B/F, LPS, TNF-α, GLP-1, and lipids. Results showed a reduction in pro-inflammatory markers and LPS with vitamin D supplementation. Therefore, targeting the gut microbiota with vitamin D could be an attractive strategy in the treatment of diabetes [76]. It has been shown that vitamin D supplementation was associated with beneficial effects on gut microbiota composition. This was observed in normal-weight and overweight groups [76]. In the normal weight group, it induced a decrease in several microbiotas, i.e., Erysipelotrichaceae, Turicibacteraceae, and Bifidobacteriaceae. The same effect was also observed among overweight participants, in addition to a decrease in pathogenic bacteria (Actinomycetaceae) [76]. Furthermore, vitamin D, through epigenetic remodeling, regulates intestinal physiology. Approximately 3% of the mouse and human genomes are regulated directly or indirectly by vitamin D signaling [77].

Microbiota composition is also influenced by vitamin D status. Vitamin D supplementation not only increased the microbiota diversity but also increased the B/F. It is well established that high Firmicutes and low Bacteroidetes are associated with MetSyn and obesity. Furthermore, investigators found increased beneficial microbes such as Bifidobacterium and Akkermansia [78]. In contrast, in children, the same investigators found that vitamin D deficiency caused an overabundance of Bacteroidetes, resulting in a significantly higher B/F compared to those who were vitamin D sufficient [63]. Perhaps the disparity in the findings between these 2 studies can be attributed to the differences in study subjects. The B/F is known to play a key role in maintaining intestinal homeostasis and is regarded as a determinant of gut dysbiosis. Therefore, vitamin D deficiency leads to dysbiosis, a probable reason for increased vulnerability to inflammatory-mediated illnesses such as MetSyn, T2DM, and obesity.

## 7. Limitations

Limitations of current literature include heterogeneity in studies with different study populations, vitamin D assessment methods, experimental protocols, and variability in outcome measures specifically with the gut microbiome. Other limitations include the nature of MetSyn as being a multifactorial disorder that needs to be demonstrated in a more holistic approach, in addition to the difficulty in extrapolating studies on the gut microbiota of rodent models to humans.

## 8. Conclusions

In conclusion, evidence supports a three-way axis between vitamin D, microbiota, and cardiometabolic diseases. An adequate level of vitamin D had a beneficial effect on gut microbiota. Dysbiosis related to low vitamin D has been associated with the risk of developing cardiometabolic diseases such as MetSyn, obesity, and T2DM. People with obesity and diabetes have altered gut microbiome. Attention should be given to the role of vitamin D and microbiota in MetSyn as this is an established precursor for T2DM and other cardiometabolic diseases. Further research is needed to clarify the intricate link between vitamin D, microbiota, and cardiometabolic diseases. Although evidence is accumulating for the supplementation of vitamin D as an adjuvant treatment for cardiometabolic diseases, clearer evidence from large-scale, well-controlled studies are still needed.

## Figures and Tables

**Table 1:** Summary of randomized control trials on the effect of serum vitamin D concentrations and biomarkers of cardiometabolic diseases ^1^.

Reference	Study Design	Study Sample & I/C	Dose and Duration of Vitamin D Supplementation	Outcome Measurements	Findings Related to Cardiometabolic Diseases ^2^	Other Findings ^2^
Cojic et al. [8]	RCT	Subjects with T2DM *n* = 130 (65/65)	Vitamin D deficient: 50,000 IU/week vitamin D, first 3 months and 14,000 IU/week, next 3 months Vitamin D sufficient: 14,000 IU/week, 6 months	FBG, HDL-C TG, WC, BP, HbA1c	Effect of time on FBG and BP was significant ↓HbA1c	
Javed et al. [38]	Double-blind RCT	Women with PCOS *n* = 37 (18/19)	3200 IU/day vitamin D, 3 months	BP, FBG, TG, HDL-C, CRP	↓HOMA-IR, ALT, E LF score, Hyaluronic acid	
Krivoy et al. [39]	Double-blind RCT	Subjects with schizophrenia *n* = 47 (24/23)	14,000 IU/week, 8 weeks	WC, HDL-C, TG, FBG, SBP	No significant difference between treatment and placebo groups	↑total MoCA score (cognitive performance)
Yin et al. [40]	RCT	Vitamin D deficient *n* = 123 (61/62)	700 IU/day vitamin D, 12 months	FBG, TG, HDL-C, WC, BP	No significant difference between treatment and placebo groups	↓serum PTH
Talaei et al. [41]	Single arm study	Subjects with T2DM *n* = 100	50,000 IU/week vitamin D, 8 weeks	FBG, HDL-C, insulin, HOMA-IR	↓FBG, insulin, HOMA-IR	-
Salekzamani et al. [42]	Double-blind RCT	Subjects with MetSyn *n* = 71 (35/36)	50,000 IU/week vitamin D, 16 weeks	FBG, HDL-C, TG, WC, BP	↓TG	-

^1^ Abbreviations: 25(OH)D, 25-hydroxyvitamin D; ALT, Alanine transaminase; BP, Blood pressure; ELF, Enhanced liver fibrosis; FBG, Fasting blood glucose; HbA1C, glycosylated hemoglobin; HDL-C, High-density-lipoprotein cholesterol; HOMA-IR, Homeostatic model assessment for insulin resistance; I/C, Intervention/Control; LDL-C, Low-density- lipoprotein cholesterol; MetSyn, Metabolic syndrome; MoCa, Montreal cognitive assessment; PCOS, Polycystic ovarian syndrome; PTH, Parathyroid hormone; RCT, Randomized controlled trial; SBP, Systolic blood pressure; T2DM, Type 2 diabetes mellitus; TG, Triglycerides; tHcy, Total homocysteine; TNF-α, Tumor necrosis factor-alpha; WC, Waist circumference. ^2^ ↑ Increased and/or direct association; ↓ Decreased and/or inverse association.

**Table 2:** Summary of cross-sectional studies on the relationship between serum vitamin D concentrations and biomarkers of cardiometabolic diseases ^1^.

Reference	Study Design	Study Sample	Vitamin D Assessment	Outcome Measurements: Biomarker of Cardiometabolic Diseases	Findings Related to Cardiometabolic Diseases ^2^
Ganji et al. [9]	Cross-sectional	NHANES *n* = 8241	Serum 25(OH)D	FBG, HDL-C, TG, WC, BP, MetSyn prevalence	↓DBP, TG, WC, HbA1c, serum insulin, C-peptide, CRP, tHcy, HOMA-IR, MetSyn prevalence ↑HDL-C
Liu et al. [10]	Cross-sectional	Elderly *n* = 2493	Serum 25(OH)D	FBG, HDL-C TG, WC, BP, MetSyn prevalence	↓TG, MetSyn prevalence ↑HDL-C
Raposo et al. [11]	Cross-sectional	Adults *n* = 4095	Serum 25(OH)D	FBG, HDL-C TG, WC, BP, MetSyn Prevalence	↓TG, BP
Pott-Junior et al. [15]	Cross-sectional	Older adults *n* = 265	Serum 25(OH)D	FBG, HDL-C, TG, WC, BP, MetSyn prevalence	↓MetSyn prevalence, IR
Bennouar et al. [43]	Cross-sectional	Adults with and without NAFLD *n* = 874	Serum 25(OH)D	FBG, HDL-C, TG, WC, BP, MetSyn prevalence	↓MetSyn prevalence
Yoo et al. [44]	Cross-sectional	Subjects with psychotic disorders *n* = 302	Serum 25(OH)D	FBG, HDL-C, TG, WC, BP, MetSyn prevalence	↓MetSyn prevalence, BP
Chew et al. [45]	Multi-center cross-sectional	Patients with lupus erythematosus *n* = 1163	Serum 25(OH)D	FBG, HDL-C, TG WC, BP, MetSyn prevalence	↓BP, TG, HOMA-IR, MetSyn prevalence ↑HDL

^1^ Abbreviations: 25(OH)D, 25-hydroxyvitamin D; BMI, Body mass index; BP, Blood pressure; CRP, C-reactive protein; DBP, Diastolic blood pressure; FBG, Fasting blood glucose; HbA1C, glycosylated hemoglobin; HDL-C, High-density-lipoprotein cholesterol; HOMA-IR, Homeostatic model assessment for insulin resistance; IR, insulin resistance; LDL-C, Low-density- lipoprotein cholesterol; MetSyn, Metabolic syndrome; NHANES, National Health and Nutrition Examination Survey; SBP, Systolic blood pressure; TG, Triglycerides; tHcy, Total homocysteine; TNF-α, Tumor necrosis factor-alpha; WC, Waist circumference. ^2^ ↑ Increased and/or direct association; ↓ Decreased and/or inverse association.

**Table 3:** Summary of animal and human studies on the effect of microbiota on the biomarkers of cardiometabolic diseases ^1^.

Reference	Study Design	Study Sample Characteristics	Interventions	Measurements	Findings ^2^ (Association with Microbiota)
Backhed et al. [52]	Experimental	Germ-free mice and conventionally raised mice	Conventionalization with microbiota from CONV-R	Body composition, IR	↑60% body fat, IR, hepatic TG synthesis
Fei and Zhao [53]	Experimental	Germ-free mice	LPS (endotoxin) transfer	Obesity Insulin resistance	↑Obesity, IR
Vrieze et al. [55]	Experimental	Lean (*n* = 9) and individuals with MetSyn (*n* = 9) (6 week)	Intestinal microbiota transfer	Insulin Sensitivity Large and small intestine gut microbiota composition	↑butyrate-producing intestinal microbiota, insulin sensitivity
Cani et al. [60]	Experimental	Mice	High fat diet (4 week) Subcutaneous infusion of LPS	Insulin sensitivity Oral glucose tolerance tests LPS tolerance test	↑LPS containing microbiota in the gut, circulating LPS, IR, hyperglycemia, hyperinsulinemia, weight gain
Amar et al. [64]	Longitudinal	Without diabetes or obesity at baseline (9 years of follow-up) *n* = 3280	-	Diabetes, 16S rDNA concentration	↑16S rDNA (with diabetes)
Pendyala et al. [59]	Cohort	*n* = 8	High-fat Western-style diets (1 month)	Gastrointestinal barrier function, Microbiota composition	↑Plasma endotoxins

^1^ Abbreviations: CONV-R, Conventionally raised; GI, Gastrointestinal; IR, Insulin resistance; LPS, lipopolysaccharide; MetSyn, Metabolic syndrome; rDNA, recombinant DNA; T2DM, Type-2 diabetes mellitus; TG, Triglycerides; WAT, White adipose tissue. ^2^ ↑ Increased and/or direct association; ↓ Decreased and/or inverse association.

## Data Availability

Not applicable.
